# The Effects of the 2020 BLM Protests on Racial Bias in the United States

**DOI:** 10.1177/01461672241269841

**Published:** 2024-09-11

**Authors:** Maximilian A. Primbs, Rob W. Holland, Freek Oude Maatman, Tessa A. M. Lansu, Ruddy Faure, Gijsbert Bijlstra

**Affiliations:** 1 Radboud University, Nijmegen, The Netherlands; 2 University of Groningen, The Netherlands; 3 Florida State University, Tallahassee, USA

**Keywords:** implicit bias, bias of crowds, Black Lives Matter, causal inference

## Abstract

The 2020 Black Lives Matter (BLM) protests in response to the murder of George Floyd highlighted the lingering structural inequalities faced by Black people in the United States. In the present research, we investigated whether these protests led to reduced implicit and explicit racial bias among White U.S. Americans. Combining data from Project Implicit, Armed Conflict Location Event Data Project (ACLED), Google Trends, and the American Community survey, we observed rapid drops in implicit and explicit measures of racial bias after the onset of the protests. However, both types of racial bias slowly increased again over time as (attention to) BLM faded. We use directed acyclic graphs to show under which assumptions causal inferences are warranted. We discuss our results in light of situational models of bias, their implications for protest movements, and raise questions about when and how social norms play a role in large-scale attitude change.

On May 25, 2020, American police officers killed George Floyd. A video of the incident led to nation-wide protests and rekindled the Black Lives Matter (BLM) movement. This movement aims to highlight and overcome the discrimination, racism, and systematic oppression Black people face. Here, we investigate whether, and if so, through which psychological processes, this large-scale societal movement succeeded in some of these aims and successfully reduced implicit and explicit racial bias toward Black people in the United States.

Although racial bias has traditionally been studied through individual-based perspectives (e.g., Forscher et al., 2019; Greenwald & Lai, 2020), we position our research in recent situational models of bias. These models postulate that bias does not merely exist in the minds of people (Murphy et al., 2018; Murphy & Walton, 2013) but also and perhaps more importantly is a feature of places or situations (Payne et al., 2017; Payne & Hannay, 2021). Places and situations can hereby reflect physical or geographical locations (Calanchini et al., 2022), but also organizations or systems and their rules, laws, norms, and practices (Murphy et al., 2018; Payne & Hannay, 2021). As such, situational models of bias suggest that racial bias should be conceptualized as a psychological state determined by situational factors, such as the 2020 BLM protests, more than as a trait determined by individual characteristics.

For example, recent studies have shown that people in areas with more confederate statues and higher historical dependence on slavery show higher levels of implicit bias nowadays (Payne et al., 2019; Vuletich & Payne, 2019). Crucially, and fundamentally incompatible with individual-based perspectives, higher levels of implicit bias in a given area are also associated with higher levels of behavioral discrimination by other people in the same area. That is, lower levels of faculty diversity and social mobility (Vuletich & Payne, 2019), higher levels of disproportionate use of force by police officers (Hehman et al., 2018), and greater racial disparities in traffic stops (Stelter et al., 2022; but see Ekstrom et al., 2022) are all associated with higher levels of implicit bias.

Situational models further predict that changes in the environment lead to changes in bias (Payne et al., 2017). Congruently, research on the Supreme Court decision to legalize gay marriage has been linked to faster decreases in anti-gay bias after as compared with before the decision (Ofosu et al., 2019). Moreover, the election of Donald Trump as U.S. president increased the perceived acceptability of prejudice against social groups targeted by Trump (Crandall et al., 2018), and eventually also increased racial and religious prejudice among Trump supporters (Ruisch & Ferguson, 2022). This suggests that established political institutions can shape both bias and the development of bias over time.

Evidence regarding the impact of massive social movements is less clear. A recent review of literature on long-term changes in implicit and explicit bias suggests that social movements are a parsimonious explanation for long-term attitude change (Sawyer & Gampa, 2022). The authors argue that social movements may influence attitudes by encouraging sympathy, reducing intergroup anxiety, changing media representations (see also Dunivin et al., 2022), and increasing intergroup solidarity (among other possible pathways).

Yet, empirical evidence shows mixed results. Considering BLM, while there is evidence that the 2013–2016 protests decreased racial bias (Sawyer & Gampa, 2018), research on the 2020 protests indicates rather small or no effects on public opinion and various measures of explicit prejudice (Boehmke et al., 2023; Reny & Newman, 2021; Shuman et al., 2022). Yet, nothing is known about the effects of the 2020 BLM protests on implicit bias. The 2020 BLM movement is an interesting case study because it is the most salient political protest movement in North America of the past decades. As such, if any contemporary political movement has the potential to reduce bias, it should be the 2020 BLM protests.

Therefore, we examined the effects of the 2020 BLM protests on both implicit and explicit racial bias. We expected and preregistered that the slow but steady decrease in both implicit and explicit racial bias observed before the onset of the BLM protests (Charlesworth & Banaji, 2019, 2022) will accelerate after the onset of the 2020 BLM protests (H1; Ofosu et al., 2019; Sawyer & Gampa, 2018). Importantly, we will make use of the Project Implicit database, which allows us to track day-to-day changes in bias in response to the BLM protests, providing a fine-grained analysis of the effects of societal events on implicit and explicit bias.

Previous research suggests that social norms are the underlying mechanism driving the effects of large-scale societal events on attitudes (Ofosu et al., 2019; Ruisch & Ferguson, 2022). For example, Ofosu and colleagues (2019) argued, but did not test, that legislation passed by democratic governments may be perceived as reflecting the majority opinion—that is, prevailing local social norms—which in turn influences attitudes and behavior. We argue that large-scale societal movements may influence attitudes and behavior via the same route: Ubiquitous protests, unwavering, often positive media attention, and widespread celebrity support signal strong social norms in support of the causes supported by the movement. As a result, people start aligning their personal beliefs with these new social norms leading to attitude and behavior change (Ruisch & Ferguson, 2022; but also see Tankard & Paluck, 2016, 2017 for evidence for the argument that social norms shift and influence behavior without changes in attitudes).

To test the role of social norms, we preregistered five hypotheses which should be supported if social norms were the underlying mechanism through which societal movements can change attitudes. First, we investigate norm salience (how accessible norm information is; Miller & Prentice, 2016): We expect that in states where the norms signaled by BLM are more salient—operationalised as the number of protests (H2a) and the number of Google searches for BLM (H2b)—there are larger reductions in racial bias over time. Second, we investigate in-and-out-group norms. In-group norms are more likely to guide attitudes and behavior than out-group norms (e.g., Smith & Louis, 2009; Wenzel, 2004) and BLM is more closely associated with the Democratic party compared with the Republican party. Therefore, we predict a larger reduction in racial bias over time for more Democratic compared with more Republican states (H2c) and for people who self-identify as relatively liberal compared with those who self-identity as relatively conservative (H2d). This is in line with research on the 2020 protests, which shows that the protests increased support for democratic candidates (Caren et al., 2023) and shifted attitudes predominantly among liberal people (Reny & Newman, 2021). Finally, if social norms were the underlying mechanism, changes in attitudes should mirror the trajectory over time of perceived social norms (people’s perception of how society evaluates Black people; H2e). That is, if we observe more positive implicit and explicit personal attitudes, we should also observe that people believe that society evaluates Black people more positively.

## Methods

### Data Sets and Measures

We obtained measures of implicit and explicit racial bias, perceived social norms, political orientation, gender, and age from the 2020 race IAT data set from Project Implicit (Xu et al., 2022). As we are testing questions related to social norms, we want to ensure that all participants have the same racial in-group. Therefore, we restricted analyses to completed sessions of White participants based in the United States (see Ofosu et al., 2019, who apply the same logic to sexual minorities). Moreover, to allow for the inclusion of gender as a control variable we removed participants who reported more than one gender identity. Finally, in line with standard IAT scoring algorithms (Greenwald et al., 2003), we removed participants with 10% or more responses faster than 300 ms, leaving a total sample of up to 428.855 participants. We combined this data set with various other data sources, each described in detail below.

#### Implicit Bias

Implicit bias was measured with the evaluative race implicit association test (IAT; Greenwald et al., 1998). In this IAT, participants categorize faces as belonging to African Americans (henceforth referred to as Black people) or European Americans (henceforth referred to as White people) and words as being positive or negative words (e.g., “excellent,” “evil”). Typically, White participants are faster and more accurate in trials where White faces and positive words share a key and in trials where Black faces and negative words share a key, compared with the reversed combination of categories. As such, higher IAT D scores are interpreted as higher levels of implicit racial bias toward Black people (Greenwald et al., 2003). Although the validity and the reliability of the IAT to measure individual differences in implicit biases still is debated in the field (e.g., Carpenter et al., 2023; Connor & Evers, 2020), recent work has established the validity and the reliability of the IAT to implicit biases at various levels of aggregation (Hehman et al., 2019).

#### Explicit Bias

Explicit racial bias was measured with feeling thermometers (Wilcox et al., 1989). Participants were instructed to rate how warm or cold they feel toward White and Black people on a 11-point Likert-type scale ranging from (1) “Extremely Cold” to (11) “Extremely Warm.” Besides using these two ratings separately, we calculated an explicit bias score by calculating the difference score (Black minus White). As such, more positive difference scores reflect a stronger preference for White people compared with Black people.

#### Perceived Social Norms

Perceived social norms were measured by calculating an aggregate score of up to six perceived norm questions answered on a 9-point Likert-type scale. For example, participants were asked: “How warm or cold does the average person feel toward Black people” or “How much does the average person like or dislike Black people.”

#### Political Orientation

Individual-level political orientation was measured by asking participants to self-identify on a 7-point Likert-type scale ranging from (1) “strongly conservative” to (7) “strongly liberal.” State-level political orientation was represented by the proportion of democratic votes in the 2020 presidential election as published by the U.S. House of Representatives (Office of the Clerk U.S. House of Representatives, 2021).

#### Number of Protests

The number of BLM protests per state was obtained from the Armed Conflict Location Event Data Project (ACLED) database, which provides an overview of all protests in the United States in the year 2020 (Kishi et al., 2021).

#### Number of Google Searches

The proportion of Google searches for the exact term “Black Lives Matter” per state in the year 2020, relative to the total number of searches in that state, was obtained from Google Trends (Google Trends, n.d.).

#### Individual-Level Control Variables

We included participants’ age and self-reported gender identity as control variables on the individual-level. Both individual-level and state-level control variables were included to align with past research (e.g., Ofosu et al., 2019) and to account for demographic changes over time.

#### State-Level Control Variables

As control variables on the state level, we included income, employment rates, population density, and education levels. All variables were obtained from the American community survey (United States Census Bureau, 2022). Income was represented by the 5-year estimate of the median household income. Employment rates were represented by the 5-year estimate of the proportion of unemployed people. Population density was defined as the average population per square mile. Educational levels were represented by the 5-year estimate of the population aged 25 and older with a high school degree or higher.

### Analyses

We report both preregistered, confirmatory, and not preregistered, exploratory analyses. Our confirmatory analyses tested our preregistered hypotheses for implicit and explicit bias separately. The confirmatory models were mixed linear models which included time (days in 2020; that is, Time 1 would be January 1, Time 20 would be January 20), onset of the protests (before/after), number of protests per state (H2a), number of Google searches per state (H2b), political orientation of the state (H2c), and of the individual (H2d), all control variables, the fitting interactions, and a random intercept for state. Moreover, we conducted otherwise identical confirmatory analyses with perceived social norms as the dependent variable (H2e). The code below specifies the model run for implicit bias:

IAT_Score ~ Time*BeginProtests*NumberProtests + Time*BeginProtests*GoogleSearches + Time*BeginProtests*proportionDemocraticVotes + Time*BeginProtests*politicalid + age + genderIdentity+ StateIncome + StatePopulation + StateEmployment + StateEducation + (1|STATE).

The corresponding pre-registration can be found on the Open Science Framework (OSF; https://osf.io/dcgv2/?view_only=d45a31dd306045ce9e5718378e169e66). We further explored the trajectory of explicit racial bias by running additional, otherwise identical, models with the explicit evaluation of White people and Black people as dependent variables. Finally, we conducted several exploratory tests to more closely inspect a visually present drop in the dependent variables right after the onset of the protests. To that end, we compared all dependent variables directly before and after the onset of the protests. These exploratory models included onset of the protests (before/after), all control variables, and a random intercept for state. To test the robustness in these nonpreregistered analyses, we systematically varied the number of weeks included in the analysis from 1 to 4 weeks and report the results of all analyses. The code below specifies the model for implicit bias: IAT_Score ~ BeginProtests + age + genderIdentity + StateIncome + StatePopulation + StateEmployment + StateEducation + (1 | STATE). Finally, we conducted additional exploratory analyses to address potential changes in demographics as an explanation for our findings. For all analyses, we centered our continuous predictor variables. The analysis code, data, and an R Markdown document containing the results can be found on the OSF (https://osf.io/hq2tj/?view_only=580ca5f68d48405c92e21b9b112dece3). We also provide full correlation matrices and robustness analyses on the OSF. The software packages used to prepare and analyze the data can also be found on the OSF. We want to explicitly state that we did not test H2(6) from the preregistration, as this turned out to be less relevant considering the observed drop in implicit bias.

## Results

### Implicit Bias

#### Confirmatory Analyses

**Figure 1.**
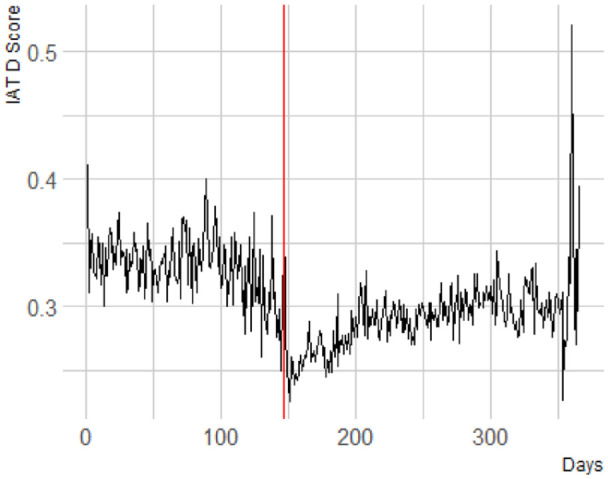
The Red Line Indicates the Onset of the Protests. The Large Increase at the End of the Year is December 24, Christmas Eve. Days Refers to the Days That Have Passed in the Year 2020.

Our results indicate that there was a significant change in the trajectory of implicit bias (i.e., a significant interaction between time and onset of protests) as recorded by Project Implicit after the onset of the 2020 BLM protests, H1; *b* < .001, *F*(1, 407,322.69) = 55.89, *p* < .001. Simple slopes revealed that there was a decrease in implicit bias over time before the onset (*b* = −.0001) and an increase after the onset (*b* = .0002). Further investigating this counterintuitive finding—we had hypothesized a stronger decrease over time after compared with before—we found that there was a drop^1^ in implicit bias right after the onset of the protests (Figure 1).

#### Exploratory Analyses

We investigated this drop further by directly comparing the mean level of bias in the weeks before the onset of the protests to the weeks after. Considering the explorative nature of these analyses, we took a mini-multiverse approach (Primbs et al., 2022; Steegen et al., 2016) and varied the time window for the comparison from 1 to 4 weeks. The data set for 1 week thus entails all participants who completed the IAT the week before and the week after the protests’ onset, whereas the data set for 2 weeks is comprised of both the week 1 data set and an additional week before and after.

We found that there was a significant drop in implicit bias after the onset of the protests for a time window before and after of 1 week, *b* = .02, *F*(1, 10,052.76) = 10, *p* = .002; 2 weeks, *b* = .03, *F*(1, 35,590.82) = 56.14, *p* < .001; 3 weeks, *b* = .03, *F*(1, 59,146.95) = 98.33, *p* < .001; and of 4 weeks, *b* = .03, *F*(1, 82,862.99) = 163.70, *p* < .001. Table 1 summarizes the test results of all exploratory analyses aimed at investigating the drop.

**Table 1.** Estimated Marginal Means, Standard Errors, Cohen’s *d* and *p*-values for Each Dependent Variable by Week (of the Exploratory Drop Analyses).

DV	Week	*M(SE)*_before_	*M(SE)*_after_	Cohen’s *d*	*p*
Implicit	1	0.204 (0.036)	0.170 (0.356)	0.081	.002
Bias	2	0.233 (0.022)	0.180 (0.021)	0.126	<.001
	3	0.246 (0.018)	0.188 (0.018)	0.134	<.001
	4	0.258 (0.016)	0.195 (0.016)	0.146	<.001
Explicit	1	−0.787 (0.135)	−0.893 (0.131)	0.068	.008
Bias	2	−0.673 (0.080)	−0.744 (0.076)	0.047	.005
	3	−0.644 (0.065)	−0.741 (0.063)	0.065	<.001
	4	−0.603 (0.059)	−0.715 (0.057)	0.075	<.001
White	1	6.37 (0.164)	6.36 (0.159)	0.002	.935
	2	6.52 (0.097)	6.52 (0.093)	0.001	.968
	3	6.51 (0.080)	6.50 (0.078)	0.009	.472
	4	6.59 (0.072)	6.53 (0.069)	0.030	.004
Black	1	7.16 (0.154)	7.26 (0.150)	−0.575	.025
	2	7.20 (0.916)	7.27 (0.088)	0.0397	.018
	3	7.16 (0.075)	7.24 (0.072)	0.0449	.001
	4	7.19 (0.067)	7.24 (0.065)	0.0298	.009
Social	1	4.02 (0.333)	3.83 (0.307)	0.190	.171
Norms	2	3.95 (0.318)	3.70 (0.304)	0.248	.008
	3	3.54 (0.329)	3.32 (0.319)	0.203	.006
	4	3.58 (0.325)	3.33 (0.318)	0.225	<.001

### Explicit Bias

#### Confirmatory Analyses

**Figure 2.**
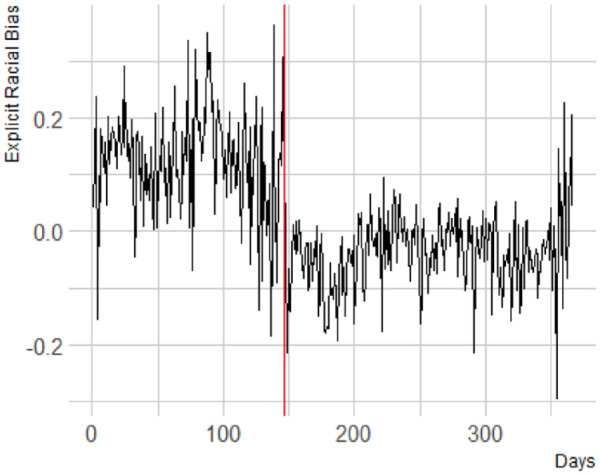
Explicit Racial Bias. A More Positive Score Indicates a More Positive Explicit Evaluation of White People Compared with Black People.

Next, we conducted our preregistered analyses for explicit bias and found no significant change in the trajectory of explicit bias after the onset of the 2020 BLM protests, H1; *b* < .001, *F*(1, 404,569.50) = 1.36, *p* = .244. However, similar to the results for implicit bias, there was a very distinct visual drop in explicit bias (Figure 2), which was significant for the full data set, before versus after; *b* = .03, *F*(1, 404,569.28) = 8.35, *p* = .004.

#### Exploratory Analyses

To further investigate the drop, we again conducted a multiverse analysis comparing the weeks before the onset of the protests to the weeks after, and showed that there was a significant drop in explicit bias for a time-window before/after of 1 week, *b* = .05, *F*(1, 10185.15) = 7.03, *p* = .008; 2 weeks, *b* = .04, *F*(1, 3,643,637) = 7.83, *p* = .005; 3 weeks, *b* = .05, *F*(1, 59,730.56) = 22.68, *p* < .001; and 4 weeks, *b* = .06, *F*(1, 83,073.17) = 42.27, *p* < .001.

### Explicit Evaluations

**Figure 3.**
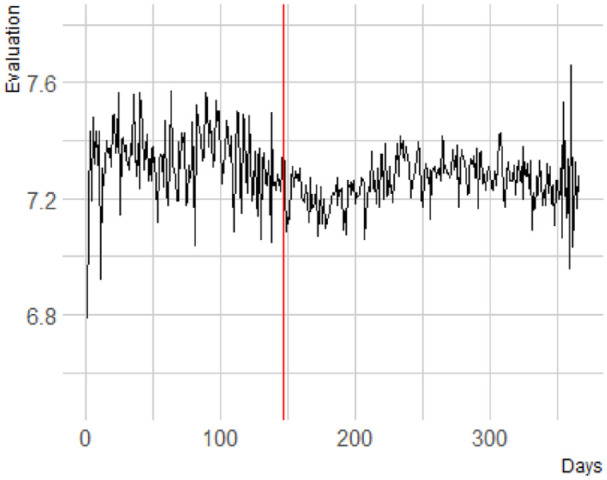
Explicit Evaluation of White People. A More Positive Score Reflects a Warmer Evaluation.

We followed up on our analyses of explicit bias by analyzing both components of explicit bias, the explicit evaluations of White people and the explicit evaluations of Black people, separately. All these exploratory analyses in this section are identical to the confirmatory analyses reported above, except for the dependent variables. Explicit evaluations of White people show a significant change in trajectory, *b* < .001, *F*(1, 404,734.09) = 5.93, *p* = .015, after the onset of the 2020 BLM protests, with a decrease in evaluation before (*b* = −.0004) and an increase after (*b* = .0001; Figure 3).

Furthermore, we investigated whether there is a change in the mean level explicit evaluation of White people before and after the onset of the protests, again employing a multiverse approach comparing 1 to 4 weeks before and after the onset. We found no evidence for a drop for 1 week, *b* < .001, *F*(1, 10,457.77) = .01, *p* = .935; 2 weeks, *b* < .001, *F*(1, 36,394.17) = 0, *p* = .968; 3 weeks, *b* = .01, *F*(1, 59,882.52) = 0.52, *p* = .472, but a significant drop for 4 weeks, *b* = .01, *F*(1, 83,064.49) = 8.43, *p* = .004.

**Figure 4.**
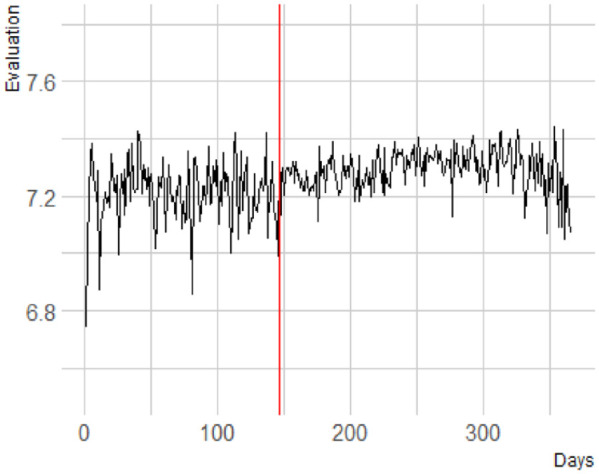
Explicit Evaluation of Black People. A More Positive Score Reflects a Warmer Evaluation.

Explicit evaluations of Black people showed a significant change in trajectory after the onset of the 2020 BLM protests, *b* < .001, *F*(1, 404678.94) = 12.13, *p* < .001. Like the explicit evaluation of White people, there was a decrease in evaluation before the onset of the protests (*b* = −.0001), and an increase in evaluation after the onset of the protests (*b* = .0005). Visual inspection (Figure 4) reveals that there seems to be an increase in evaluation after the onset of the protests.

Next, we directly compared the mean evaluation of Black people in the period before the onset of the protests to the period after the onset. Employing a multiverse approach, we found that there is a significant increase in evaluation for 1 week, *b* = −.05, *F*(1, 10,365.30) = 5.00, *p* = .025; for 2 weeks, *b* = −.03, *F* (1, 36,045.12) = 5.56, *p* = .018, for 3 weeks, *b* = −.04, *F*(1, 59,449.23) = 10.95, *p* = .001; and for 4 weeks, *b* = −.03, *F* (1, 82,812.66) = 6.75, *p* = .009.

### Social Norms

#### Confirmatory Analyses

Finally, we investigated whether the change in trajectory of bias could be explained by a shift in social norms. We tested the five preregistered hypotheses which would support social norms as a potential underlying mechanism. First, we tested whether states with more protests showed a larger change in trajectory (H2a) and found no such influence for implicit bias, *b* < .001, *F*(1, 406,692.47) = 0.51, *p* = .473, explicit bias, *b* < .001, *F*(1, 404,483.92) = 0.61, *p* = .436, the explicit evaluation of White people, *b* < .001, *F*(1, 404,516.98) = 1.59, *p* = .207, or the explicit evaluation of Black people, *b* < .001, *F*(1, 404,180.63) = 0.49, *p* = .486. Second, we tested whether there was a larger change in trajectory in states with more interest in BLM—measured by the number of Google searches (H2b)—and found no evidence for this hypothesis for implicit bias, *b* = < .001, *F*(1, 405,861.62) = .04, *p* = .846, explicit bias, *b* < .001, *F*(1, 404,326.11) = 0.53, *p* = .466, explicit evaluations of White people, *b* < .001, *F*(1, 404,197.95) = 2.02, *p* = .155, but a statistically significant effect on explicit evaluations of Black people, *b* < .001, *F*(1, 403,546.77) = 4.26, *p* = .039; given the enormous sample size and the fact that we only observe this on one of our DVs, we do not interpret this interaction further. Third and fourth, we tested whether social norms signaled by the in-group caused larger changes in trajectory and expected larger changes in trajectory for more democratic states (H2c) and people who self-identify as more liberal (H2d). On the state level, we found no evidence for such an effect for implicit bias, *b* < .001, *F*(1, 405,694.24) = 0.99, *p* = .320, explicit bias, *b* < .001, *F*(1, 404,267.58) = 0.20, *p* = .657, explicit evaluations of White people, *b* < .001, *F*(1, 404,095.77) = 1.91, *p* = .167, or explicit evaluations of Black people, *b* < .001, *F*(1, 403,362.66) = 3.16, *p* = .076. On the person-level, we also found no evidence for the hypothesized effect on explicit bias, *b* < .001, *F*(1, 404,537.08) = 3.13, *p* = .077, explicit evaluations of White people, *b* < .001, *F*(1, 404,710.34) = 1.37, *p* = .242, or explicit evaluations of Black people, *b* < .001, *F*(1, 404,699.47) = 0.03, *p* = .860, but a statistically significant effect on implicit bias, *b* < .001, *F*(1, 407,391.50) = 5.30, *p* = .021.

**Figure 5.**
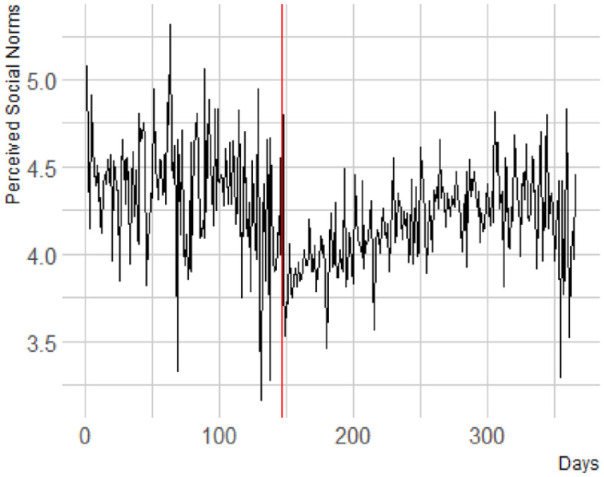
Perceived Social Norms. A Higher Score Reflects a More Positive Perceived Evaluation of Black People by Society

Finally, we investigated whether perceived social norms would show a similar pattern of change over time as racial bias (H2e). We found a significant change in trajectory of perceived social norms after compared with before the onset of the 2020 BLM protests, *b* < .001, *F*(1, 14453.78) = 12.60, *p* < .001, with a decrease before the onset (*b* = −.001) and an increase afterwards (*b* = .001). Visual inspection of the data reveals a drop in perceived social norms after the onset of the protests (Figure 5).

#### Exploratory Analyses

Further investigating the drop, we find that there was a significant decrease in perceived social norms for 2 weeks, *b* = .13, *F*(1, 1,281) = 7.04, *p* = .008; 3 weeks, *b* = .11, *F*(1, 2,118) = 7.49, *p* = .006; and 4 weeks, *b* = .12, *F*(1, 2,867.10) = 13.08, *p* < .001, but not for 1 week, *b* = .09, *F*(1, 346) = 1.88, *p* = .171. The full results tables can be found on the OSF.

### Robustness Analyses

In our preregistration we wrote that we would analyze our hypotheses H2a–d in the same model, including all relevant predictors as covariates. However, it could be that these additional predictors and interaction terms weaken the test of the other hypotheses by decreasing power. These additional analyses included each of the predictors (Number of Protests, Google Searches, State-level political orientation, and individual-level political orientation) separately (i.e., one per analysis), the control variables, a random intercept for state, and either implicit bias or explicit bias as the dependent variable. The results of these analyses confirm the results of the full model reported in detail above. Moreover, we acknowledge that our chosen set of covariates is only one possible set out of multiple reasonable sets of covariates. Therefore, we have added multiple appendices with alternative sets of covariates included in the models. First, we included a model with Ethnicity and Education on the individual-level as additional covariates. Second, we included a model with Education, but without state-level employment as covariates. Third, we included a model without any covariates. The results of all three alternative model specifications confirm the findings of our main analyses, showing that the findings are robust to covariate choices. We included the full regression tables for each of these additional analyses in the online supplementary materials (https://osf.io/hq2tj/?view_only=580ca5f68d48405c92e21b9b112dece3).

### Causal Inference

The present research makes use of existing, cross-sectional data sets with daily data collection and investigates the effect of a natural intervention: An event that randomly happened during the collection of the data (Muñoz et al., 2020). However, as most research using these Project Implicit data sets, we want to draw causal inferences about the effects of the event—in our case BLM protests—on bias (Jimenez et al., 2021; Ofosu et al., 2019). In the following section, we will discuss under which assumptions we can draw causal inferences from our data and present additional analyses to support our claims. To identify valid causal estimates, two key assumptions need to hold: Temporal ignorability and excludability (Muñoz et al., 2020). Temporal ignorability here means that the sample before and after the BLM protests is comparable on all relevant measures. Excludability here means that time does not affect implicit bias through any event except through the BLM protests.

#### Temporal Ignorability

To assess temporal ignorability, we investigated whether the sample demographics before and after the onset of the 2020 BLM protests were comparable. We assessed changes in age, gender, and political orientation, as these are the demographic variables available on Project Implicit and have been shown to be correlated to implicit bias (Charlesworth & Banaji, 2021). After the onset of the protests, participants on average were considerably older (*M*age = 39.71, *SD*age = 15.38) compared with before the onset of the protests (*M*age = 32.21; *SD*age = 13.66), *t*(117743) = −130.88, *p* < .001. Moreover, the gender distribution of participants remained virtually unchanged (before: 33% men, 65% women, 1% gender nonconforming, 1% other; after: 33% men, 66% women, 1% gender nonconforming). Finally, the sample self-identified as slightly more liberal after the onset of the protests (*M* = 5.06, *SD* = 1.73) compared with before the onset of the protests (*M* = 4.63, *SD* = 1.75), *t* (107034) = −60.887, *p* < .001. This means that the temporal ignorability assumption is violated regarding age and political orientation. Furthermore, we want to acknowledge that the assumption may be violated regarding unobserved variables.

#### Excludability

We have concluded that the sample demographics before and after the onset of the 2020 BLM protests are different. Previous research has established that demographic variables such as age, gender, and political orientation are correlated with (implicit) bias. That means, there is an alternative causal path for the effect of the protests on implicit bias: The protests may have changed the demographic composition of the sample, which in turn led to the observed decrease in implicit bias. In other words, the violation of the excludability assumption (i.e., BLM changed who visits Project Implicit) is causing a violation of the temporal ignorability assumption (i.e., the sample demographics changed), which opens up a backdoor path and an alternative explanation. To not violate the assumption of excludability and to rule out this backdoor path, we must correct for all demographics or other covariates that influence implicit bias and could explain the observed decrease in response to the event; and demonstrate that there were no other simultaneous events or random fluctuations in implicit bias causing this observed decrease. As there were no simultaneous high-profile events directly relevant to implicit bias beyond those that could be directly related to or considered part of the BLM protests (such as the murder of George Floyd itself), we believe we can exclude the possibility of a simultaneous relevant event. Similarly, the observed change seems to surpass observed “normal” fluctuations in observed implicit bias levels within Project Implicit.

#### Directed Acyclic Graphs

**Figure 6.**
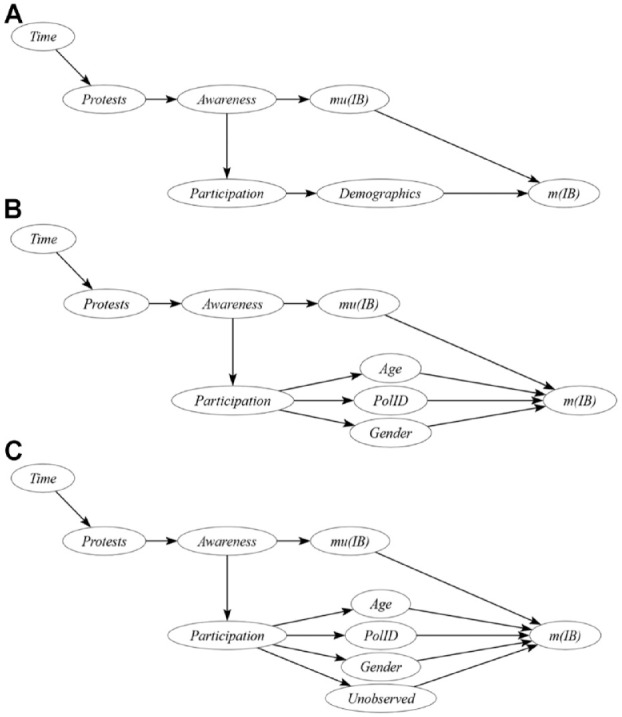
Panel A Depicts Our Full Theoretical Model. Panel B Depicts the Restricted Model Which Assumes That Age, Gender, and Political Orientation are the Only Demographic Variables That Need to be Accounted for. Panel C Depicts an Alternative Model Which Allows for the Presence of Unobserved Variables That Need to be—but Cannot be—*Accounted for.*

To summarize, the observed decrease in implicit bias may either be a consequence of the protests directly, or a consequence of changes in the demographic composition of the sample. We use directed acyclic graphs (DAG; Rohrer, 2018) to display this causal model and present alternative models which address the violation of the assumptions (Figure 6). DAGs are a visual representation of causal assumptions and thus aid researchers with thinking more clearly about the relationships between variables. We point the interested reader to Rohrer (2018) for a very detailed introduction to DAGs for psychological research.

Panel A displays our full theoretical causal model for implicit bias. The model for explicit bias would be identical. Time is represented here as a cause of the protests due to our data structure; time is the variable by which the protests’ presence varies and is an important source of potential alternative explanations (see also the preceding section on “Temporal ignorability”). We argue that the murder of George Floyd and the subsequent protests led people to become aware of the BLM movement. Awareness of the protests, and the norms and values it signals, in turn caused a decrease in the population mean of implicit bias, which is reflected in a decrease in the observed sample mean of implicit bias. However, both the ignorability and the excludability assumption are violated, which means that we cannot exclude alternative paths through which the protests may have changed the observed sample mean. After awareness of the protests increased, the type of people visiting the Project Implicit website may have changed, leading to a change in sample demographics which also could have led to a change in the observed sample mean of implicit bias, while the population mean could have remained stable or changed in another direction. In Panel B, we present a restricted causal model that assumes that the observed demographic variables age, gender, and political orientation are the only sample demographic variables that could have relevantly affected the observed mean implicit bias in such a way. Finally, in Panel C, we present an alternative causal model that allows for the presence of other, unobserved yet causally relevant variables.

#### Restricted Model

We conducted additional tests with the aim to draw causal inferences from a restricted model that assumes that the observed demographic variables age, gender, and political orientation are all variables that need to be accounted for to control for self-selection biases driving the observed mean change. We found that the sample on average becomes more liberal and older after the onset of the protests. Age is positively related to implicit bias, meaning that an increase in age cannot explain a decrease in bias. However, more liberal people generally show lower levels of implicit bias. This entails that our observed decrease in racial bias might be explained by the increased proportion of liberal people completing the IAT.

**Figure 7.**
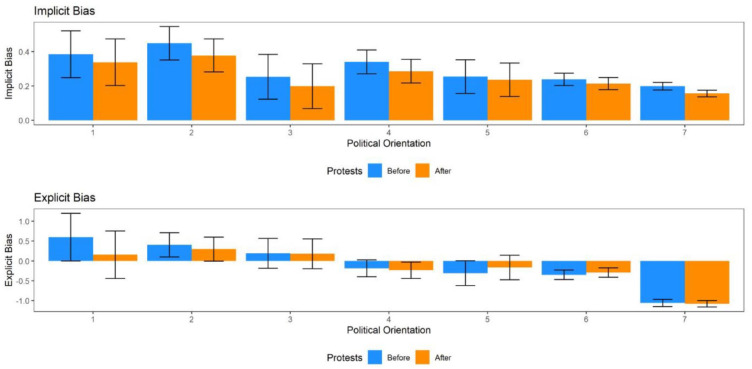
Drop by Political Orientation for Implicit and Explicit Racial Bias. 1 = “Strongly Conservative,” 7 = “Strongly Liberal”.

Therefore, we conducted additional tests investigating the drops in racial bias after the onset of the BLM protests within each scale point of the political orientation scale. For sub-samples for each scale point, we assessed whether there was a drop of comparable magnitude as in the full sample. We manually compared the observed standardized effect sizes. We explicitly want to highlight that we do not evaluate the statistical significance of these findings, given the vast discrepancies in sample sizes between analyses. Like in the full sample, we again employed a multiverse approach comparing time spans ranging from 1 week to 4 weeks before and after the beginning of the 2020 BLM protests (Figure 7).

For *strongly conservative* individuals, we found evidence for a drop in mean level implicit bias for 3 weeks (*d* = 0.121, *p* = .140) and 4 weeks (*d* = 0.117, *p* = .090). Moreover, we found evidence for a drop in explicit bias for 1 week (*d* = 0.242, *p* = .165), 2 weeks (*d* = 0.224, *p* = .034), 3 weeks (*d* = 0.299, *p* <.001), and 4 weeks (*d* = 0.249, *p* = <.001). For *moderately conservative* individuals, we found evidence for a drop in implicit bias for 2 weeks (*d* = 0.133, *p* = .015), 3 weeks (*d* = 0.176, *p* < .001), and 4 weeks (*d* = 0.174, *p* < .001). In addition, we found evidence for a drop in explicit bias for 3 weeks (*d* = 0.053, *p* = .224) and 4 weeks (*d* = 0.085, *p* = .021). For *slightly conservative* individuals, we find a drop in implicit bias for 3 weeks (*d* = 0.092, *p* = .041) and for 4 weeks (*d* = 0.130, *p* = .001), but no evidence for drops in explicit bias. For politically *neutral* individuals, we find a drop in implicit bias for 2 weeks (*d* = 0.146, *p* < .001), 3 weeks (*d* = 0.132, *p* < .001), and 4 weeks (*d* = 0.129, *p* < .001), but no evidence for drops in explicit bias. For *slightly* and *moderately liberal* individuals, we find no evidence for drops in either implicit or explicit bias. For *strongly liberal* individuals, we find evidence for a drop in implicit bias for 1 week (*d* = 0.103, *p* = .079), 2 weeks (*d* = 0.132, *p* = .001), 3 weeks (*d* = 0.117, *p* < .001) and 4 weeks (*d* = 0.098, *p* < .001), but no evidence for drops in explicit bias. We provide the full analysis results and additional analyses in the online supplementary materials (https://osf.io/hq2tj/?view_only=580ca5f68d48405c92e21b9b112dece3).

Here, we aimed to establish causal effects of the 2020 BLM protests on implicit and explicit bias in a restricted model that assumes gender, age, and political orientation are all demographic variables that need to be accounted for. We found that after correction there was a drop only in two of the seven political orientation categories for explicit bias. The drop in explicit bias is only present in the two most conservative sub-groups, which also have the highest mean level of explicit bias before the onset of the protests. That means, the absence of a drop in explicit bias in the other, beforehand less biased groups, could simply reflect a floor effect. If people’s explicit biases are already small, it is hard to reduce them even further.

However, for implicit bias five of the seven political orientation categories (all but slightly and moderately liberal) show evidence of a drop right after the onset of the BLM protests.

To obtain stronger evidence for causality under Model B, we conducted propensity score weighting with the WeightIt (version 0.14.2; Greifer, 2023b) and the cobalt (version 4.5.3; Greifer, 2024) packages. Propensity score weighting is a type of propensity score analysis, an approach to causal inference for observational data very popular in the medical sciences (e.g., Ye & Kaskutas, 2009). It allows researchers to obtain unbiased causal estimates of treatment effects when treated and untreated subjects differ systematically on important covariates (see Austin, 2011; Barrett et al., 2024; Greifer, 2023a for introductions). In other words, by applying propensity score weighting, we can ensure that the samples before and after the 2020 BLM protests are similar on the observed covariates. Here, we implemented propensity score weighting using inverse probability weights and the average treatment effect as causal estimand (see Greifer & Stuart, 2023 for an introduction). For each week (Weeks 1–4), we assessed balance using standardized mean differences and Kolmogorov-Smirnov statistics (see OSF for full results). If the samples were still unbalanced, we used entropy balancing instead (Hainmueller, 2012). After, we estimated a weighted random effect model with implicit or explicit bias as dependent, and Begin of Protests, age, gender, and political orientation as independent variables, a random intercept for state, and the propensity scores as weights. Followingly, we used a procedure called weighted G-Computation (Snowden et al., 2011; Vansteelandt & Keiding, 2011) with cluster-robust CR2 standard errors (Pustejovsky & Tipton, 2017) implemented in clubSandwich (version 0.5.10; Pustejovsky, 2023) to estimate unbiased marginal treatment effects from those models (Greifer, 2023a). Table 2 shows the treatment effects and the means for the propensity score models.

**Table 2.** Propensity Score Models.

DV	Week	ESS	*M(SE)*_before_	*M(SE)*_after_	Estimate	*p*
Implicit	1	10,774.29	0.276 (0.127)	0.264 (0.007)	−0.012	.325
Bias	2	37,110.42	0.280 (0.009)	0.252 (0.004)	−0.028	.002
	3	60,189.11	0.297 (0.007)	0.255 (0.004)	−0.031	<.001
	4	88,378.31	0.293 (0.006)	0.258 (0.003)	−0.034	<.001
Explicit	1	10,774.29	−0.131 (0.035)	−0.029 (0.023)	−0.016	.678
Bias	2	37,110.42	−0.030 (0.032)	−0.018 (0.019)	0.012	.719
	3	60,189.11	−0.026 (0.027)	−0.027 (0.013)	−0.002	.950
	4	88,378.31	−0.022 (0.028)	−0.030 (0.014)	−0.008	.749

*Note.* ESS stands for effective sample size. The ESS is an estimate of the size an unweighted sample would need to have to have the same precision as our weighted sample. The ESS reflects the fact that there is loss in precision because of the weighting (Greifer, 2023a).

If gender, age, and political orientation indeed are all variables that need to be accounted for (Panel B), this study demonstrates that the 2020 BLM protests caused drops in implicit bias but not in explicit bias.

#### Alternative Model

The restricted model assumes that gender, age, and political orientation are all variables that need to be accounted for. This is a strong assumption—we know from the literature that implicit bias is influenced by many different factors, some of which may also be affected by the onset of the protests. For example, the protests could have led to a greater influx on the Project Implicit website of people on both sides of the political spectrum who have high levels of concern for Black people. Likewise, there could be selection on implicit bias itself unrelated to changes in other sample demographics. This change in an unobserved demographic variable could have then caused the drop in implicit bias. The model in Panel C therefore allows for the presence of such an unobserved demographic variable, which causes the observed drop.

## Discussion

In the present research, we investigated the effects of the 2020 BLM protests on implicit and explicit racial attitudes in the United States. Inconsistent with H1, we found that BLM did not accelerate the decrease of racial bias. Instead, we observed a rapid drop in implicit and explicit racial bias directly after the onset of the protests. Moreover, we showed that under the strict assumptions highlighted above, we can argue that the effect of the protests on implicit but not explicit bias is causal.

The observed decrease in racial bias after the onset of the 2020 BLM protests partially aligns with past research. Specifically, studies found that the 2020 BLM protests increased support for police reforms (Reny & Newman, 2021; Shuman et al., 2022) and shifted the public discourse surrounding racism (Dunivin et al., 2022). Yet, while our results on explicit bias align with previous work showing limited changing in explicit attitudes in response to BLM (Reny & Newman, 2021; Shuman et al., 2022), we observed considerable drops in implicit racial bias right after the onset of the protests.

There are two possible explanations for this drop: First, the 2020 BLM protests could have caused the drop. This would align with situational models of implicit bias, which predict that changes in the social and cultural environment should lead to changes in implicit bias (Figure 6, Panel A; Payne et al., 2017). Initially, we had expected that this change in bias would take place gradually over time—as it did in response to Trump’s presidential election (Charlesworth & Banaji, 2022). Instead, we observed an abrupt drop in implicit racial bias immediately after the onset of the protests. This is consistent with research showing that single events, if extreme and highly diagnostic, can change implicit attitudes (Cone & Ferguson, 2015; Ferguson et al., 2019). We argue that the murder of George Floyd constitutes such an extreme and highly diagnostic event and confronted millions of Americans with counter-attitudinal information, triggering rapid changes in implicit bias.

Second, as depicted in Figure 6, an alternative explanation for the observed drop could be that Project Implicit attracted different people after the onset of the 2020 BLM protests compared with before; and that this change of sample composition caused the (rapid) drop in bias. Given that the sample composition indeed changed after the protests, we also tested this alternative explanation. Using DAGs (Rohrer, 2018) and ancillary analyses we showed that in a restricted causal model that assumes age, gender, and political orientation are all variables that could have caused this drop independently from a population change in implicit bias, we can still identify a causal effect of the protests on implicit bias. Yet, we cannot exclude that a change in sample composition on unmeasured, further variables could be driving the drop. This suggests that we need more knowledge about potential causal structures surrounding the concept of implicit bias to draw strong conclusions from nonexperimental data.

Regardless of which explanation you favor, the phenomenon that the Project Implicit website attracted different people is interesting on its own: Why did the onset of the 2020 BLM protests change the demographic composition of the Project Implicit sample (see also Sawyer & Gampa, 2018)? One explanation is that the IAT and hence Project Implicit was promoted more strongly in particular groups or contexts, where people from particular backgrounds that tend to score low on implicit bias are present (e.g., highly educated groups). Another explanation is that people who heard of the concept of implicit bias before were intrigued what their implicit bias scores were given the situation at the time (i.e., the protests). This opens an interesting avenue for future research.

Moreover, we found no evidence that social norms are the mechanism underlying the observed changes in bias. Neither the number of protests (H2a), the number of Google searches (H2b), nor in-group effects on a state (H2c) or individual level (H2d) showed the predicted effect. This can be explained on a methodological level, as each of the measures of social norms used in H2a–H2d had some shortcoming. One study suggests that the effect of the BLM protests themselves on the public opinion were geographically very limited—to a few miles around the protest location (Boehmke et al., 2023). This suggests that the state-level operationalization we used as organizing principle for H2a–H2c may not fine-grained enough. Moreover, the number of protests was statistically conflated with the murder of George Floyd, as there were many more protests after compared with before the murder. Concerning the use of Google Trends in H2b, Google Trends data only assesses the relative frequency of searches for “Black Lives Matter” and does not allow inferences about people’s motivation for the search. That means, two states could have a very high relative frequency of searches but could differ very strongly in average motivation for the search.

Importantly, the measure with the highest face validity, people’s perceptions of how society evaluates Black people (i.e., perceived social norms), does show an effect: People’s perceptions of how society evaluates Black people becomes more negative right after the onset of the protests. This is contrary to what we predicted: As BLM aimed to highlight the oppression faced by Black people, massive protests in support of BLM should have triggered the descriptive norm that people care about equality. However, the fact there are massive protests against racism to begin with, signals that racism is widespread and must be protested—it signals a different descriptive social norm. In fact, many of the signs displayed by BLM protestors (“Stop killing us”; MacArthur & Ritter, 2020) explicitly signal that there is a widespread problem with racism in the United States. While we intended to measure the first social norm, it is entirely conceivable that we instead measured the second social norm. This is further supported by the fact that other researchers have also used this measure as a direct measure of prejudice (Hester et al., 2023), casting doubt on what exactly it measures. Therefore, operationalizing social norms as originally intended with this measure may not be conceptually appropriate.

Together, our findings indicate that there is no evidence that the observed change in racial bias after the 2020 BLM protests is caused by changes in social norms, as originally hypothesized. The mechanism through which protests influence bias may therefore be different from the mechanism through which Supreme Court decisions influence bias (Ofosu et al., 2019). Alternatively, it is plausible that different types of social norms related to race were activated simultaneously, partially counteracted each other or were not picked up by our measures. Future research should attempt to explain this finding, for example by investigating what types of descriptive norms are activated by societal protests, how different types of norms relate to implicit bias, how to best measure social norms activated by social events, and examine some of the other potential mechanisms through which protests may change attitudes (as outlined in Sawyer & Gampa, 2022).

We explicitly want to contextualize the effect sizes observed in the present research.

Direct comparisons of the time periods before and after the protests yielded effect sizes ranging from *d* = .081 to *d* = .146 for implicit bias. Judged on common (Cohen, 1988) or empirically derived (Lovakov & Agadullina, 2021) standards, these effect sizes would be “small.” However, when compared with other effect sizes from similar studies (e.g., Ofosu et al., 2019), our effects are considerable: After the Supreme Court decision to legalize gay marriage, there is a decrease of .011 in the IAT D score per year. In contrast, 4 weeks after the onset of the BLM protests implicit bias had decreased by an IAT D score of .060. That means, the effect of the Supreme Court decision to legalize gay marriage would need to persist for almost 6 years to decrease implicit bias by the same amount as BLM did in 4 weeks .

A limitation of the present research is the nonrepresentativeness of the Project Implicit data. As noted by previous research, this data is usually not representative of the U.S. population, as people providing data through the website are younger, more likely to be female, and more liberal (e.g., Ofosu et al., 2019), and more importantly often self-selected into the website. However, as argued by Ofosu and colleagues (2019) because of the observed relationships between Project Implicit data and various meaningful population outcomes (for a review, see Calanchini et al., 2022), there seems to be meaningful variation in the data. Moreover, given frequently raised concerns about data collected from undergraduate samples at Western universities, as is done in most psychological studies, the Project Implicit data is still more representative of the general population than most data sets used in psychological research (Henrich et al., 2010).

To conclude, in the present research we provided novel evidence showing rapid drops in implicit (but not explicit) racial bias after the onset of the 2020 BLM protests. Yet, the decrease did not sustain over time—showing a different pattern than previous research on the 2013–2016 protests (Sawyer & Gampa, 2018)—and a different pattern than research that investigated the consequences of a Supreme Court decision (Ofosu et al., 2019). The death of George Floyd and the resulting immediate reactions were followed by a rapid decrease in racial bias, but these changes were short-lived. For long-term changes in the social and cultural environment—and corresponding long-term changes in attitudes and behavior—protests may be insufficient but might need to be translated into policy and legislative changes that have stronger potential to permanently alter existing social norms (Tankard & Paluck, 2017).

## Notes

1. Technically a drop also implies a (very brief) change in trajectory in the hypothesized direction. However, that was not the type of change in trajectory we expected. Therefore, we do not view this as support for our hypotheses.
